# Path Planning and Impedance Control of a Soft Modular Exoskeleton for Coordinated Upper Limb Rehabilitation

**DOI:** 10.3389/fnbot.2021.745531

**Published:** 2021-11-01

**Authors:** Quan Liu, Yang Liu, Yi Li, Chang Zhu, Wei Meng, Qingsong Ai, Sheng Q. Xie

**Affiliations:** ^1^School of Information Engineering, Wuhan University of Technology, Wuhan, China; ^2^School of Electronic and Electrical Engineering, University of Leeds, Leeds, United Kingdom

**Keywords:** path planning, rehabilitation robot, impedance control, coordinated rehabilitation, soft exoskeleton

## Abstract

The coordinated rehabilitation of the upper limb is important for the recovery of the daily living abilities of stroke patients. However, the guidance of the joint coordination model is generally lacking in the current robot-assisted rehabilitation. Modular robots with soft joints can assist patients to perform coordinated training with safety and compliance. In this study, a novel coordinated path planning and impedance control method is proposed for the modular exoskeleton elbow–wrist rehabilitation robot driven by pneumatic artificial muscles (PAMs). A convolutional neural network-long short-term memory (CNN-LSTM) model is established to describe the coordination relationship of the upper limb joints, so as to generate adaptive trajectories conformed to the coordination laws. Guided by the planned trajectory, an impedance adjustment strategy is proposed to realize active training within a virtual coordinated tunnel to achieve the robot-assisted upper limb coordinated training. The experimental results showed that the CNN-LSTM hybrid neural network can effectively quantify the coordinated relationship between the upper limb joints, and the impedance control method ensures that the robotic assistance path is always in the virtual coordination tunnel, which can improve the movement coordination of the patient and enhance the rehabilitation effectiveness.

## Introduction

In China, the lifetime risk of stroke is 39.9%, ranking first globally. Almost 85% of stroke survivors have difficulties with upper limb motor functions (Liu et al., 2020). The human upper limb participates in a wide variety of activities of daily living (ADL) tasks, and its damage will seriously reduce the mobility and quality of life of patients. The injury of the upper limb will be reflected in the abnormal inter-joint coordination (Brokaw et al., 2014). Stroke patients, especially in the late stage of recovery, tend to have abnormal movement problems in the elbow and wrist while the shoulder joint has a relatively lower possibility of injury (Bilić et al., 2001; Squeri et al., 2014). Patients after stroke tend to suffer from upper limb dysfunction, such as the loss of coordination or being unable to perform coordinated movements. The recovery of upper limb coordination is essential for stroke patients to improve their prognosis (Saita et al., 2020). Robot-assisted therapy can provide patients with highly intensive training to intensify their motor function (Hsieh et al., 2018) and help recover their limb movement coordination (Carpinella et al., 2020).

Upper limb rehabilitation robots can be divided into two types, end-effectors or exoskeletons (Zhang et al., 2018). Compared with end-effectors, exoskeleton robots can assist patients in multiple joints which is more suitable for the human body (Zimmermann et al., 2019). It is worth noting that traditional rehabilitation robots are usually driven by rigid motors, which bring problems such as high stiffness and lack of compliance (Dindorf and Wos, 2020). In contrast, pneumatic artificial muscle (PAM) is lightweight and compliant, which meets the requirements of both safety and comfort during rehabilitation (Ghobj et al., 2017). More recently, Santos et al. (2017) developed a modular lower limb rehabilitation exoskeleton composed of telescopic tubular and modular structures. Garrido et al. (2016) designed the 4-degree of freedom (DOF) modular upper limb exoskeleton. As modular robots can be assembled into a variety of configurations to adapt to different requirements and working conditions, it has become a trend to design rehabilitation exoskeletons with soft and modular structures.

Coordination is the ability to maintain context and dependent relationships between different body segments or joints (Broome et al., 2019). To perform coordinated training, it is necessary to conduct a quantitative analysis of the coordination relationship of upper limb joints. There are some studies on the quantitative coordination analysis of upper limbs in the task space (van Dokkum et al., 2014; Johansson et al., 2017). However, as the upper limb is redundant, the coordination in the task space cannot guarantee that the joint space is coordinated as well (Tomita et al., 2017). Thus, joint coordination must be investigated. In previous studies, principal component analysis (Tang et al., 2019), artificial neural networks, and other methods (Fineman and Stirling, 2017) have been used to quantify the coordination of upper limb joints. With coordination quantification, exoskeletons can be controlled to assist human limbs by following the coordination law. In this way, patients can undergo more coordinated and natural training.

Accurate position control is the basis of various robot-assisted training modes. Pneumatic artificial muscles have been adopted in some rehabilitation robots. Model-based (Huang et al., 2018; Wang et al., 2019) and model-free methods (Tu et al., 2017; Giancarlo, 2019) have been proposed to tackle nonlinear and hysteretic characteristics. Considering the difficulties in PAM modeling, model-free methods have been proved to be more advantageous. During robot control, the purpose is to regulate the position and/or speed to assist human joints to achieve coordinated training (Proietti et al., 2016). Progress in kinematic coordination has been made in lower limb rehabilitation (Gui et al., 2017), such as space robots (Lu and Jia, 2019), dual-arm surgical robots (Wu et al., 2019), and bilateral robots (Miao et al., 2020), but there are few examples in upper limb rehabilitation. Brokaw et al. (2013) implemented a time-independent functional training mode on the ARMin III robot, to realize the coordinated training for stroke patients. Crocher et al. (2012) transformed the tracking problem from the task trajectory space to the joint velocity space to realize special coordination among joints. Proietti et al. (2017) also contributed to the coordinated control of upper limb robots.

The current coordinated control of robots is mainly based on force control. When the movement of joints deviates from the reference trajectory, the robot will generate a torque to drive the limb back to the desired trajectory. During this process, however, safety problems may be caused by the interaction force. In addition, the reference trajectories are usually a set of arbitrary curves without following the coordination laws. Maintaining the active intention of the patient while improving movement coordination is crucial in rehabilitation practice. The joint angles specified by one's own data can motivate participation of patients and generate personalized paths. Machine learning methods have been recently used in joint angle prediction. Gholami et al. (2020) chose the convolutional neural network (CNN), Feiyun et al. (2015) proposed the multiple linear regression autoregressive model and Kalman filter (MLRAR-KF), and Xie et al. (2020) took the artificial neural network (ANN), to predict the joint angles. The coordination relationship between different joints and the control compliance should be fully considered. The existing coordination trajectories cannot adapt to different subjects or rehabilitation stages (Deng et al., 2020; Li Z. et al., 2020), while the convolutional neural network-long short-term memory (CNN-LSTM) algorithm can extract complex features and analyze continuous data by combing the advantages of CNN and LSTM, which can be used to generate personalized coordination trajectories.

The purpose of this paper is to propose a coordinated path planning and impedance control method for the elbow and wrist joints with a modular elbow–wrist exoskeleton. A soft modular elbow–wrist exoskeleton was designed for stroke patients and the neural network was used to quantify the coordinated movement of the upper limb joints. Based on the trajectory tracking control, a coordinated control method of the elbow–wrist robot based on impedance adjustment was proposed to help patients perform active training safely and compliantly with the guidance of the coordination relationship. The main contribution includes the quantification of the coordination trajectories with CNN-LSTM from the data collected under ADL tasks and the control of a compliant exoskeleton for interactive coordinated training. The rest of this paper is organized as follows: Section Soft Modular Exoskeleton demonstrates the modular elbow–wrist exoskeleton. In Section Path Planning and Impedance Control for Coordinated Upper Limb Rehabilitation the proposed coordinated control method with a joint coordination model is presented. The coordination quantification and robot control experiments are conducted in Section Experiments and Results. The discussion and conclusion are presented in Section V.

## Soft Modular Exoskeleton

Considering the movements of the elbow flexion/extension and wrist flexion/extension, the elbow angle is always greater than 0° and is equal to 180° when the arm is straight. When the palm and forearm are in a straight line, the wrist angle is equal to 0°. The wrist angle is defined as positive in flexion and negative in extension. The elbow range of motion is 30–180°, while the wrist is −30–50° for the common society. The designed elbow–wrist exoskeleton must meet such a range of motion.

**Figure 1:**
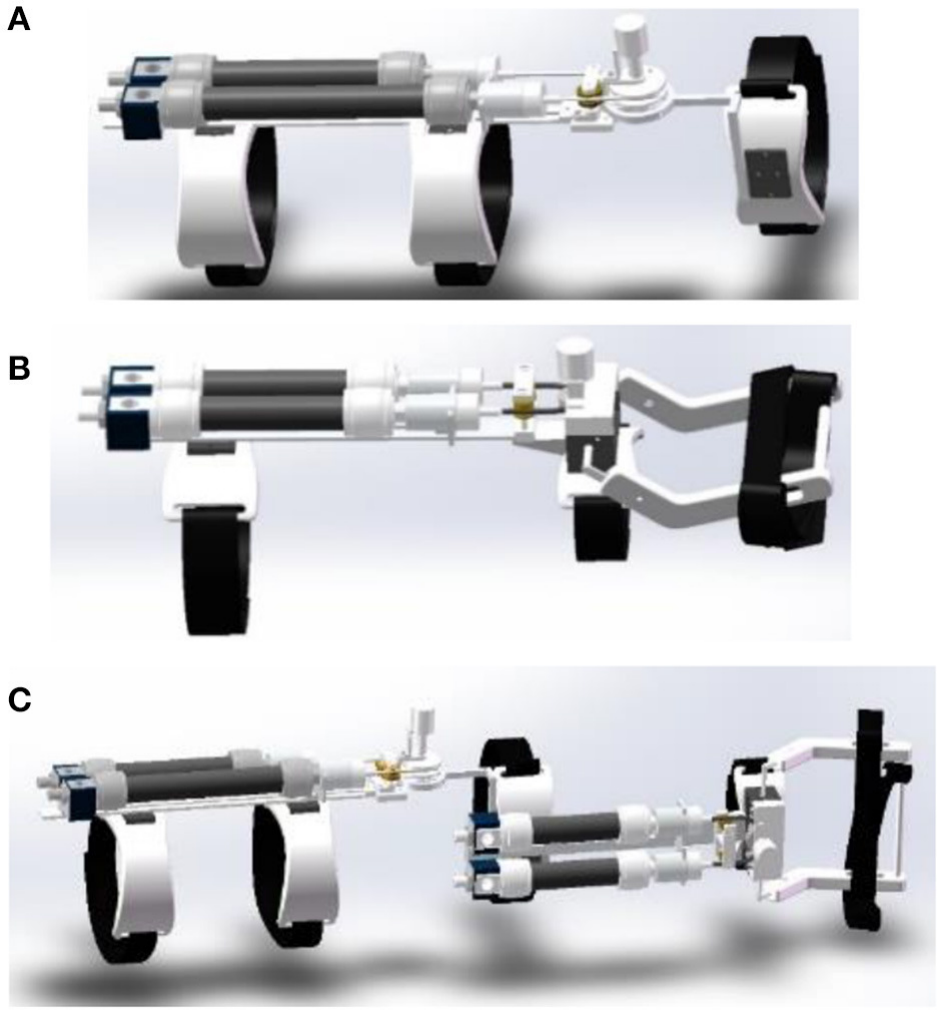
The designed soft modular elbow–wrist rehabilitation exoskeleton driven by PAMs, **(A)** the elbow module, **(B)** the wrist module, and **(C)** the reconfigured with modular parts.

The current rehabilitation robots have drawbacks in terms of their fixed structure and insufficient scalability. The modular structure can solve these problems well. Rehabilitation training using modular robots can be customized for different patients or different parts to improve the utilization of the robot. On the other hand, modular robots have distinct structures and are easier to assemble and maintain. Based on the reconfigurable concept, a modular elbow–wrist rehabilitation robot driven by PAMs was designed. The elbow/wrist modules and the reconfigured elbow–wrist exoskeleton are shown in Figure 1. A pulley was used to convert the linear motion of PAMs to the rotary motion of the joint. In the initial state, the PAMs contracted to half of the maximum contractible length. When the PAMs on both sides alternately inflate or deflate, the joint will begin to rotate under the pull rope at the end of the PAMs.

The elbow–wrist rehabilitation robot is composed of an elbow module and a wrist module, and the splicing position is located at the upper arm connecting rod and forearm PAMs bracket. The wrist module realizes different degrees of freedom rehabilitation by replacing the corresponding sub-function modules. When the connecting bolt is installed, the elbow module and the wrist module are connected, and the robot as a whole can perform rehabilitation for the elbow and wrist joint of the patient at the same time. When disassembled, the elbow module and the wrist module are separated and independent of each other. The modular design also makes the device expandable and adjustable, which can suit the coordinated rehabilitation well with the changing paths and/or forces from different joints which can work separately or together. To ensure safety and comfort during rehabilitation, other parts of the elbow–wrist exoskeleton are made of flexible materials fabricated by a three-dimensional (3D) printer to reduce the weight.

## Path Planning and Impedance Control for Coordinated Upper Limb Rehabilitation

### Coordination Tasks and Data Acquisition

**Figure 2:**
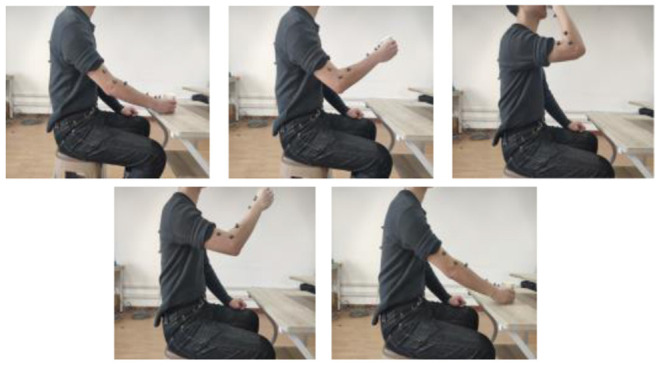
Coordinated training, Task 1: drinking water.

**Figure 3:**
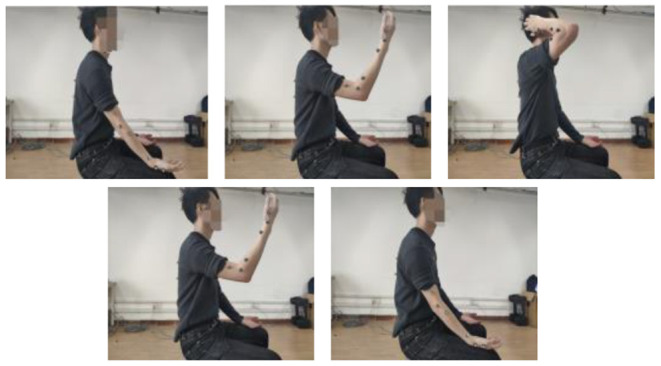
Coordinated training, Task 2: touching head.

To quantify the coordination relationship of the upper limb in joint space, we selected two representative reach and grasped tasks with moderate range, as shown in Figures 2, 3: drinking water and touching the head. Detail Task 1: a cup is placed on the horizontal table in front of the subject, and the distance is about four-sevenths of the arm length. During the task, the subject is always sitting upright while the abdomen is at the same level as the desktop. In the initial state, the subject holds the cup with the right hand, then raises the cup to the mouth and completes the drinking, finally puts down the cup and returns to the initial state. Detail Task 2: During the whole process, the sitting posture of the subject is the same as in Task 1. In the initial state, the right hand of the subject is placed on the right thigh, and the palm is upward in a horizontal relaxation state. Then the right hand is raised, reaches, and touches the head. Finally, the arm returns to its initial state. There is no limit on the speed in both tasks and each subject should avoid redundant actions. All the subjects were right-handed and have no arm disease or joint injury. The information of the subjects is shown in Table 1. The studies involving the human participants were reviewed and approved by the Ethics Committee of the Wuhan University of Technology. The patients/participants provided their written informed consent to participate in this study.

**Table 1:** Information of the recruited subjects.

**Subject**	**Gender**	**Age**	**Height (cm)**	**Weight (kg)**
S1	Male	24	178	72
S2	Male	25	169	56
S3	Male	24	175	75
S4	Female	23	158	43

**Figure 4:**
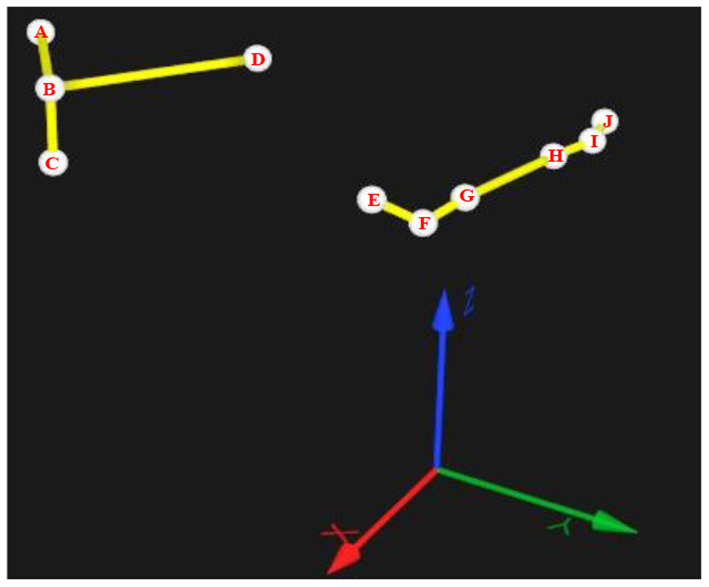
Marker points in the motion capture system and the definition of the shoulder, elbow, and wrist angles.

The motion data of the shoulder, elbow, and wrist joints when completing the tasks were collected using the motion capture system (QUALISYS Miqus M5, Sweden) with 10 marker points as shown in Figure 4. Points A, B, and C are located on the back of the subject. Point B is at the right scapula, and point C is vertically lower than point B. Point D is located on the chest, Points E, F, and G are located on the upper arm, elbow, and forearm, respectively, and points H, I, and J are located on the forearm, wrist, and hand. Thus, the shoulder angle can be represented by the angles between BA and EF, BC and EF, and BD and EF, respectively, as shoulder angle I, shoulder angle II, shoulder angle III. The Elbow angle is represented by the angle between FE and FG, and the wrist angle is represented by the angle between HI and IJ.

Then, the angle of each joint can be calculated. Take the elbow as an example, assuming that the point *E*(*x*_1_, *y*_1_, *z*_1_), *F*(*x*_2_, *y*_2_, *z*_2_), and *G*(*x*_3_, *y*_3_, *z*_3_), then


(1) $$\begin{array}{r} \vec{\text{FE}}=\left(x_{1}-x_{2},y_{1}-y_{2},z_{1}-z_{2}\right) \end{array}$$

(2) $$\begin{array}{r} \vec{\text{FG}}=\left(x_{3}-x_{2},y_{3}-y_{2},z_{3}-z_{2}\right) \end{array}$$

Then the elbow angle is


(3) $$\begin{array}{r} \beta =cos^{-1}\frac{\vec{\text{FE}}\cdot \vec{\text{FG}}}{\left|\vec{\text{FE}}||\vec{\text{FG}}\right|} \end{array}$$

Similarly, the shoulder and wrist angles can be calculated.

### Coordination Relationship Quantification

**Figure 5:**
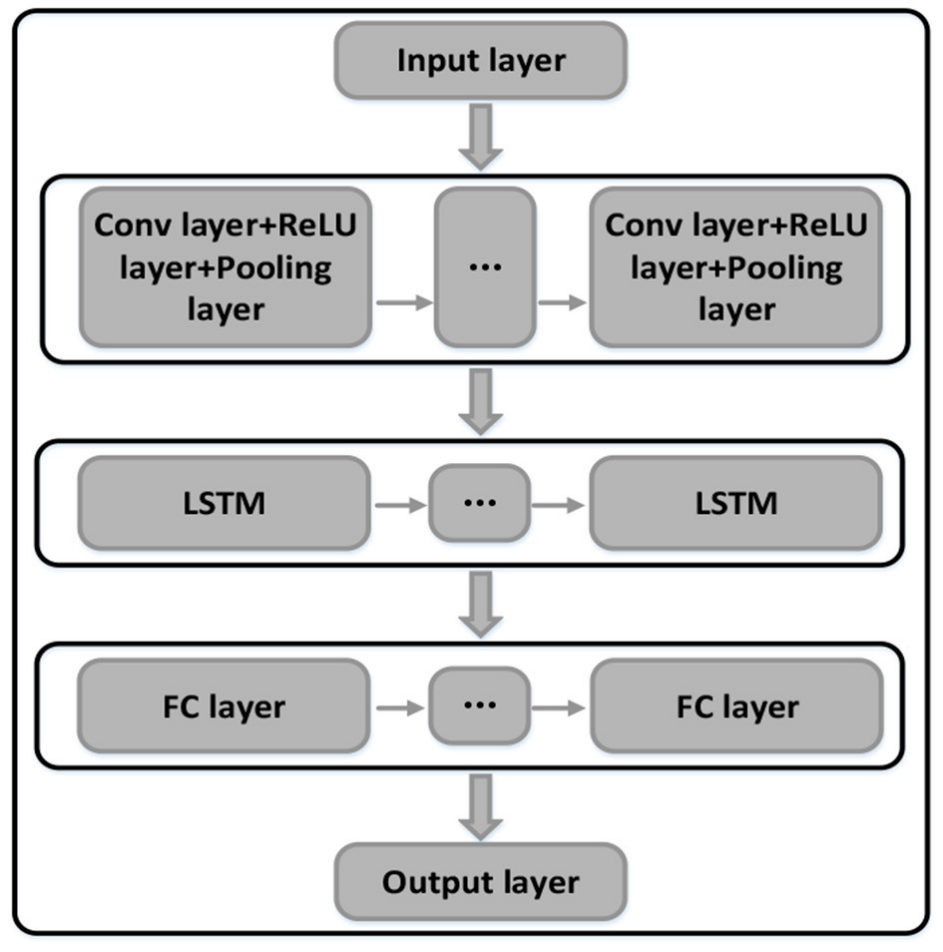
The CNN-LSTM hybrid neural network for joint coordination relationship quantification.

Due to the differences in the subjects, the pre-defined trajectories might not follow the coordination laws. The personalized coordination trajectories are thus required to meet the individual training needs. Using the recorded motion data when completing coordination tasks, we can quantify the coordination relationship of each joint. The CNN is capable of extracting complex features from massive data, and the LSTM model is used to analyze continuous sequence data. In this paper, the CNN-LSTM model was adopted to quantify the coordination relationship of the upper limb joints. The diagram of the CNN-LSTM hybrid neural network is shown in Figure 5, with the network structure and the parameters properly designed or selected to improve the overall performance, including two convolution layers, two pooling layers, two dropout layers, two LSTM layers, and two full connection layers. The dropout layer can effectively suppress the neural network overfitting. The details of the parameters set of each layer are shown in Table 2. To quantify the shoulder-elbow coordination, the angle data of shoulder I, II, III, and the elbow were inputted to the CNN-LSTM model. After training the network parameters, the coordination relationship of the shoulder and elbow joints is established. Similarly, the coordination of the shoulder, elbow, and wrist can be achieved. Then, we can quantify the coordination of other subjects.

**Table 2:** Parameters of each layer of the CNN-LSTM model.

**Layer**	**Parameters**
Conv1D(1)	Filters = 80, kernel_size = 1, padding = valid
Maxpooling1D(1)	Pool_size = 2, padding = valid
Conv1D(2)	Filters = 48, kernel_size = 1, padding = valid
Maxpooling1D(2)	Pool_size = 1, padding = valid
Dropout(1)	Rate = 0.2
LSTM(1)	Units = 32
LSTM(2)	Units = 16
Dropout(2)	Rate = 0.2
Dense(1)	Units = 64
Dense(2)	Units = 1

### Path Planning

**Figure 6:**
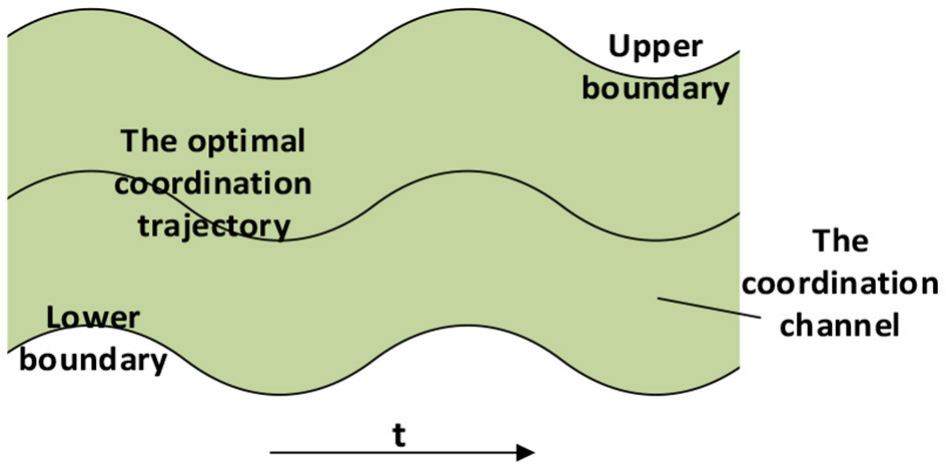
The optimal coordination trajectory and the virtual channel for the robot control ensure the motivation and coordination of patients.

The quantified results were used to generate the coordinated trajectory of the upper limb joints. The target users of this method are patients with normal shoulder movements. The data from the shoulder joint of the patient can be used to generate more personalized and coordinated elbow and wrist trajectories for rehabilitation training. The quantification was operated offline to generate a virtual channel, which was common in rehabilitation practice as the online generation may cause unexpected arbitrary trajectories harmful to patients (Deng et al., 2020; Li Z. et al., 2020). During movement, the elbow and wrist angles can be predicted *via* the CNN-LSTM model which was then used to generate the coordination trajectories. Robot-assisted training paths should be planned to the motivate participation of the patient while following the coordination laws. Patients are allowed to deviate from the desired trajectory, but the range needs to be limited. In this study, the optimal reference path is the coordination trajectory generated by the quantification results, and the coordination channel is a range around the optimal trajectory. The robot trajectory can be corrected around the coordination path according to human contact force, but it cannot exceed the boundary of the virtual channel to the main overall coordination. As shown in Figure 6, such a path planning strategy can retain the initiative of the patient while ensuring that the motion is always coordinated.

Position control is the basis for coordinated control. An improved multiple input single output model-free adaptive controller (MISO-IMFAC) is proposed for the trajectory tracking of the elbow–wrist exoskeleton. Instead of decomposing it into several single pneumatic muscles, MISO-IMFAC controls the robot as an unknown model to enhance the control robustness. The control scheme is as follows, and more details of the MISO-IMFAC can be found in our previous work (Li Y. et al., 2020).


(4) $$\begin{array}{r} u\left(k\right)=u\left(k-1\right)\\ +\frac{\rho {\hat{\emptyset }}_{c}\left(k\right)\left(Q_{1}\left(\theta_{d}\left(k+1\right)-\theta \left(k\right)\right)+Q_{2}\left(\theta_{d}\left(k+1\right)-\theta_{d}\left(k\right)\right)\right)}{\lambda +Q_{1}||{\hat{\emptyset }}_{c}\left(k\right)|{|}^{2}+Q_{2}||{\hat{\emptyset }}_{c}\left(k\right)|{|}^{2}} \end{array}$$

(5) $$\begin{array}{l} {\hat{\emptyset }}_{c}\left(k\right)={\hat{\emptyset }}_{c}\left(k-1\right)\\ \;+\frac{\eta \left(\Delta \theta \left(k\right)-{\hat{\emptyset }}_{c}^{T}\left(k-1\right)\Delta u\left(k-1\right)\right)\Delta u\left(k-1\right)}{\mu +{\left\|\Delta u\left(k-1\right)\right\|}^{2}} \end{array}$$

(6) $$\begin{array}{rcl} {\hat{\varphi }}_{i}\left(k\right)={\hat{\varphi }}_{i}\left(1\right),\;if\;{\hat{\varphi }}_{i}\left(k\right)<b\;or\\ sign\left({\hat{\varphi }}_{i}\left(k\right)\right) & \neq & sign\left({\hat{\varphi }}_{1}\left(k\right)\right),\;i=1,\cdots \;,m \end{array}$$

where ***u***(*k*) ∈ *R*^*m*^ and θ(*k*) ∈ *R*^1^ represent the input and output data at time *k* respectively. ${\hat{\emptyset }}_{c}\left(k\right)$ is the pseudo gradient (PG). The control law is obtained from (4), Equation (5) is for PG estimation, and Equation (6) is a method of PG reset. The method only applies the input and output data without modeling the PAMs, which is not sensitive to the nonlinear and time-varying characteristics of the system, so the control algorithm has strong adaptability and robustness.

### Robot Impedance Control

**Figure 7:**
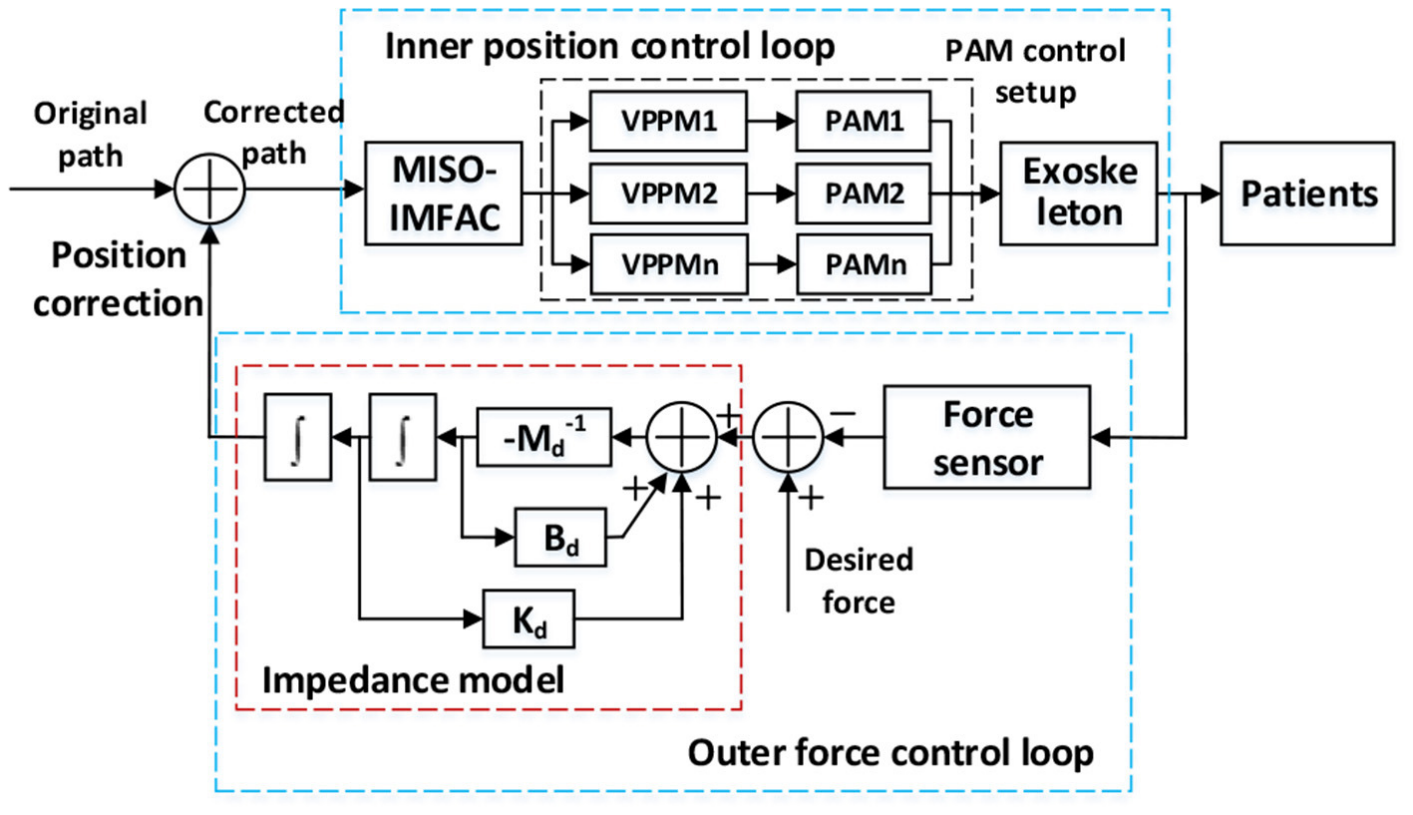
The position-based impedance control scheme for the exoskeleton with PAM control setup.

The position-based impedance control obtains the desired trajectory by mapping the error between the desired force and the actual force and corrects the final path, as illustrated in Figure 7. The impedance model can be expressed as (7).


(7) $$\begin{array}{r} M_{d}\left({\ddot{\theta }}_{d}-\ddot{\theta }\right)+B_{d}\left({\dot{\theta }}_{d}-\dot{\theta }\right)+K_{d}\left(\theta_{d}-\theta \right)=F-F_{d} \end{array}$$

where *M*_*d*_, *B*_*d*_, *K*_*d*_ represent inertia parameters, damping, and stiffness. θ, $\dot{\theta }$, $\ddot{\theta }$ represent the actual angle, angular velocity, and angular acceleration of the robot, and θ_*d*_, ${\dot{\theta }}_{d}$, ${\ddot{\theta }}_{d}$ are the desired angle, angular velocity, and angular acceleration. *F*_*d*_ is the desired contact force, and *F* is the actual contact force.

Generally for coordinated path control, the desired contact force can be assumed as *F*_*d*_ = 0, Equation (7) can be written as:


(8) $$\begin{array}{r} M_{d}\left({\ddot{\theta }}_{d}-\ddot{\theta }\right)+B_{d}\left({\dot{\theta }}_{d}-\dot{\theta }\right)+K_{d}\left(\theta_{d}-\theta \right)=F \end{array}$$

Figure 7 shows the position-based impedance (admittance) control scheme for the exoskeleton with the PAM control setup. The position correction is generated from the force loop and the original trajectory was corrected by the human interaction. Once the corrected path was received from the impedance model, the MISO-IMFAC algorithm was adopted to calculate the required displacement and control command of each PAM. Then the PAM can be controlled by tuning the air pressure inside *via* proportional valve regulators (VPPM-6L-G18-0L6H, FESTO, Germany) to drive the robot to track the corrected path. More details of the PAM control setup on a testing platform can be found in our previous work (Ai et al., 2020).

In this study, the impedance control scheme was designed with the following hypothesis considering both coordination laws and the active motivation of patients. When the movement is following the optimal coordination path, the robot can be set highly compliant, so the active intention of the patient can directly correct the original path. With the increase of the distance from the optimal path, the stiffness should gradually increase, to ensure that the robot will always operate within the coordination channel. The inertia and damping parameters also need to be adjusted accordingly. To achieve this goal, assuming that the object is in the artificial potential field (APF), the target must have an attraction to the object while the obstacle must have repulsive force. Regarding the channel boundary as the obstacle and the coordination trajectory as the target, then the impedance parameter at a certain position can be adjusted with the APF method, to encourage the robot to follow the optimal path while maintaining the active freedom of the patient.

The potential function at a point can be expressed as:


(9) $$\begin{array}{r} \text{U}\left(q\right)=U_{att}\left(q\right)+U_{rep}\left(q\right) \end{array}$$

where *U*(*q*) is the potential function, *U*_*att*_(*q*) is the gravitational potential function, *U*_*rep*_(*q*) represents the repulsive potential function, and


(10) $$\begin{array}{r} U_{att}\left(q\right)=\frac{1}{2}\varsigma d^{2}\left(q,q_{goal}\right) \end{array}$$

(11) $$\begin{array}{r} U_{rep}\left(q\right)=\{\begin{array}{c} \frac{1}{2}\eta {\left(\frac{1}{D\left(q\right)}-\frac{1}{Q^{*}}\right)}^{2},D\left(q\right)\leq Q^{*}\\ 0,D\left(q\right)>Q^{*} \end{array} \end{array}$$

where ς and η represent gain parameter, *d*(*q, q*_*goal*_) and *D*(*q*) is the distance between point *q* and the target and the nearest obstacle respectively. Q^*^ is the obstacle threshold.

The adjustment of impedance parameters with APF is:


(12) $$\begin{array}{r} \{\begin{array}{c} K_{d}=K_{d0}+c_{1}U\left(q\right)\\ B_{d}=B_{d0}+c_{2}U\left(q\right)\\ M_{d}=M_{d0}+c_{3}U\left(q\right) \end{array} \end{array}$$

where *K*_*d*_, *B*_*d*_, *M*_*d*_ represent the stiffness, damping, and inertia parameters and *K*_*d*0_, *B*_*d*0_, *M*_*d*0_ are their initial values. The positive weight factors are represented by *c*_1_, *c*_2_ and *c*_3_.

**Figure 8:**
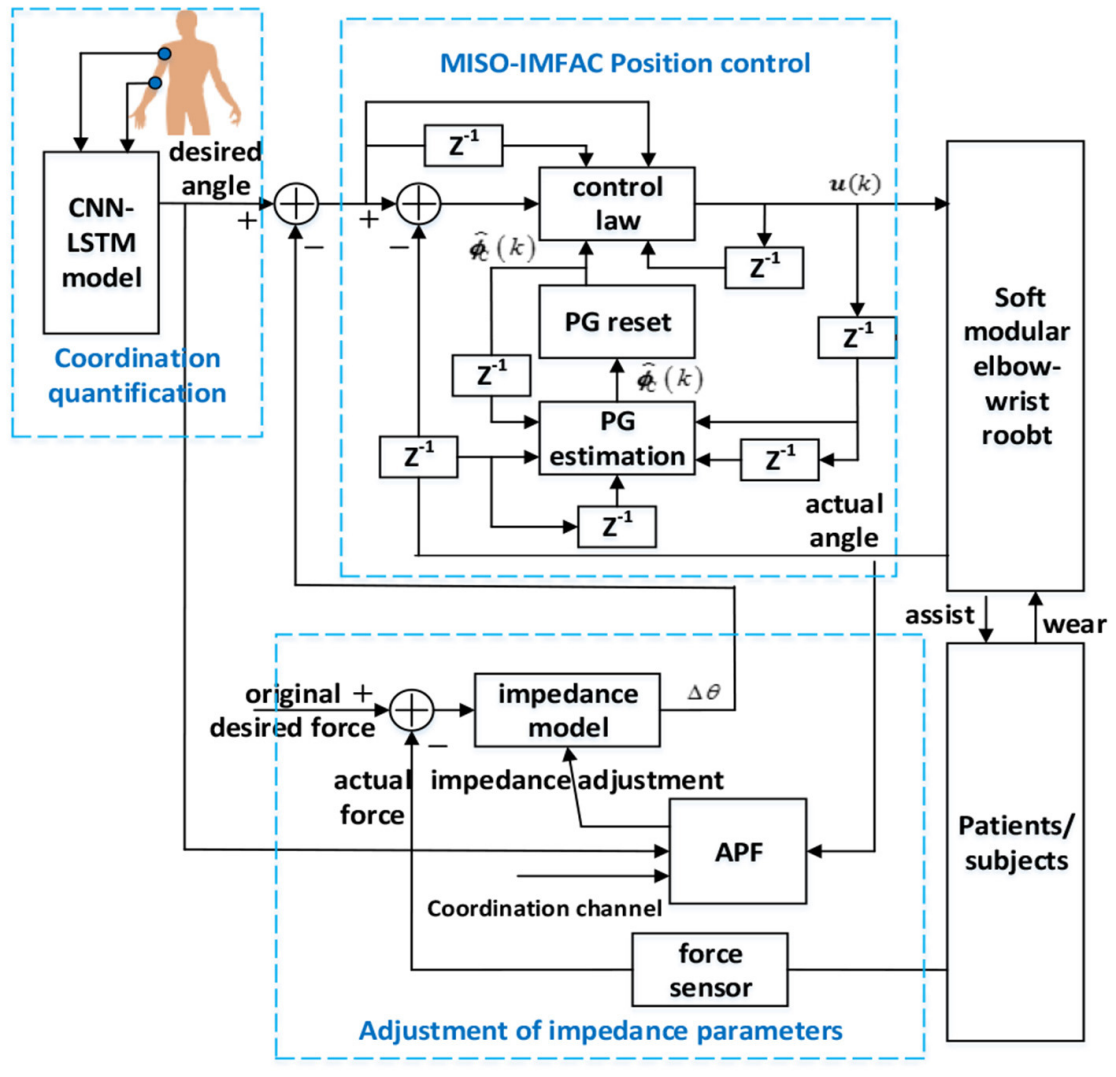
Diagram of the proposed coordinated controller with the coordination quantification, impedance adjustment, and position control.

The coordination controller of the elbow–wrist robot was designed as shown in Figure 8, which contains three parts: the coordination quantifier to generate the initial path, the impedance parameters adjuster to constrain the motion within the coordination channel, and the position controller to track the final path corrected by human contact force. For specific coordination training tasks, the elbow–wrist angle was generated *via* the CNN-LSTM model as the originally desired trajectory, i.e., the optimal coordination path. When patients exert contact force, the expected trajectory will be corrected by the impedance/admittance model tuned by the APF. Then MISO-IMFAC algorithm was used to control the robot to follow the corrected trajectory. Considering the range and compliance of the coordination channel, the APF scheme is properly set to adjust the impedance parameters of the robot to ensure the motivation and coordination of the patient during training.

## Experiments and Results

### Coordination Relationship Quantification

**Figure 9:**
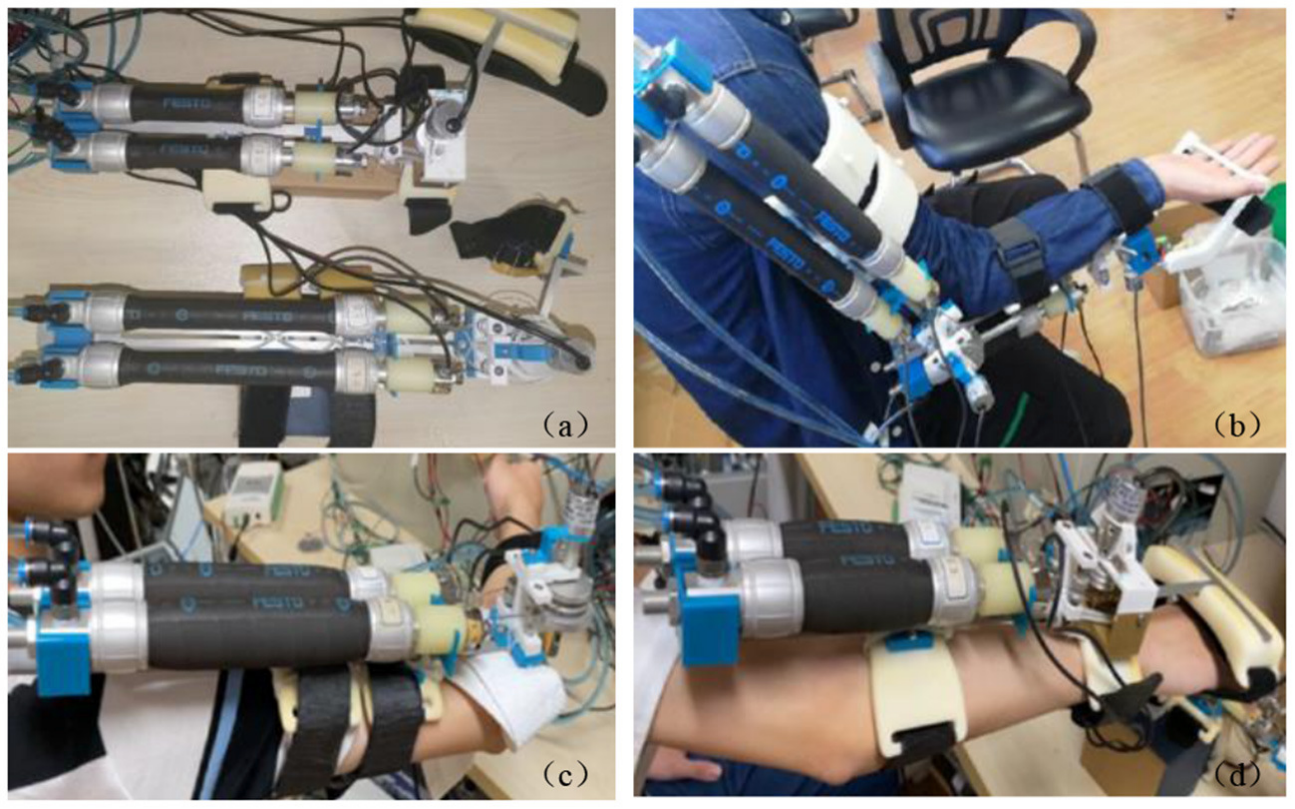
Experiment setup of the robotic exoskeleton and robot-assisted subject training: **(a)** the developed modular exoskeletons, **(b)** a subject wearing the elbow–wrist exoskeleton with modules working together, **(c)** a subject wearing the elbow exoskeleton, and **(d)** the wrist exoskeleton.

For the coordination training tasks, all the subjects wore tight clothes to prevent the change of marker positions during movement. The sampling rate of the motion capture system was 100 Hz. The experimental setup of the robotic exoskeleton and the subject wearing the exoskeleton is demonstrated in Figure 9 for the robot-assisted training. The exoskeleton has modular elbow and wrist parts that can work together or separately to meet various training requirements. Drinking water and touching the head are the two selected typical and representative ADL tasks for patient training. Each subject was instructed to perform a task within 1 min, as a trial. Each of the two tasks will be performed with four trials and a break will be taken between the two tasks. These two tasks will be repeated several times to collect enough training data on the different subjects to verify the model prediction effectiveness.

**Figure 10:**
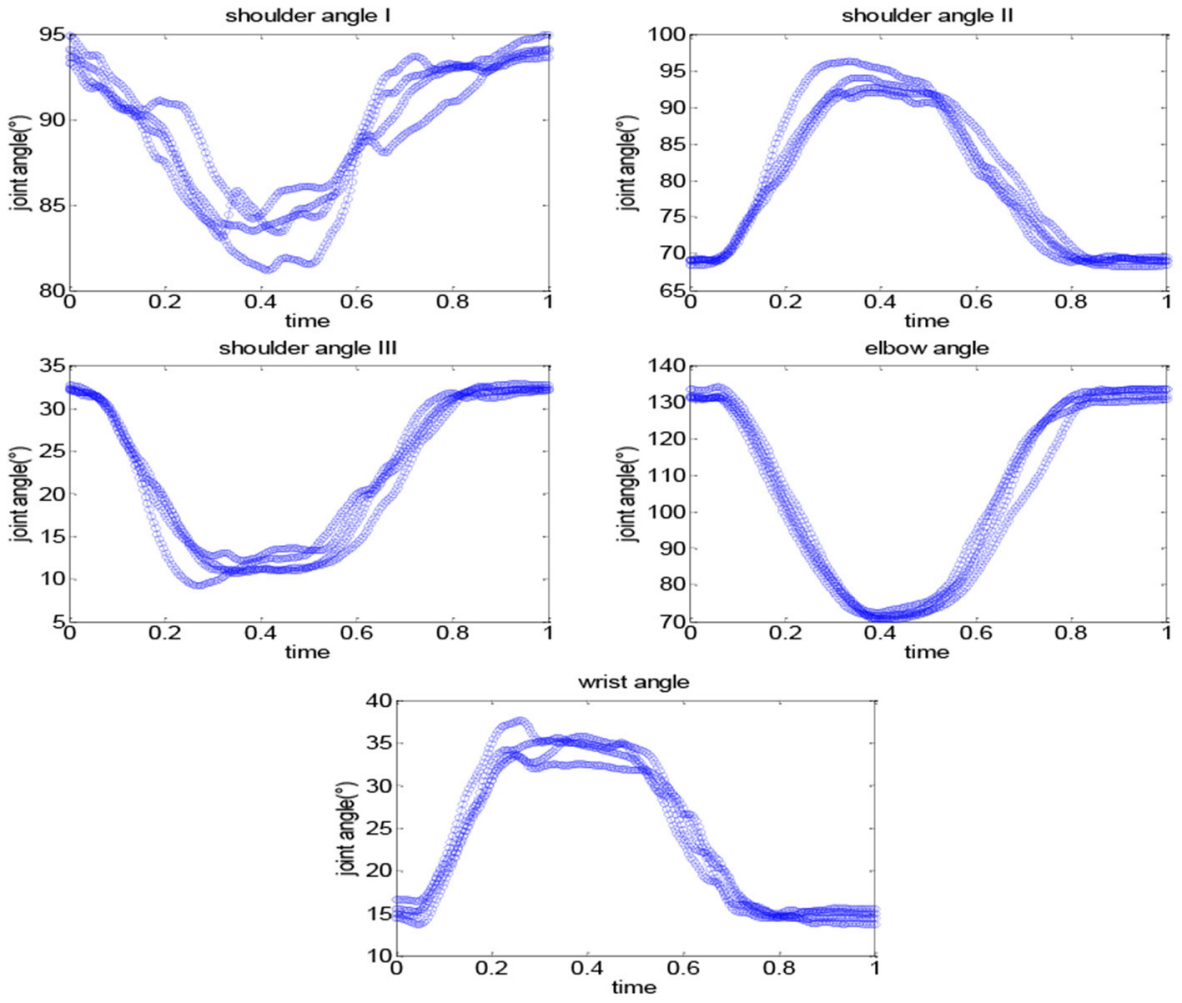
The changes of the shoulder, elbow, and wrist angles of subject S1 under Task 1: drinking water.

**Figure 11:**
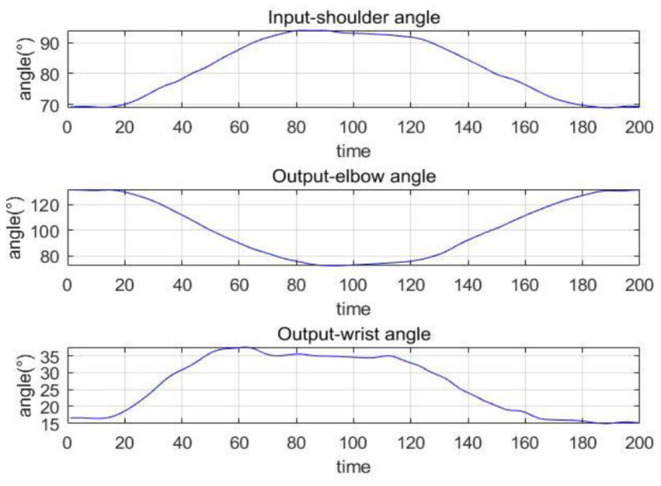
An example of the coordination relationship trained from the CNN-LSTM model (Task 1).

The collected movement data can reflect the joint angle changes during the robot-assisted movement. Figure 10 shows the changes of the shoulder, elbow, and wrist angles of subject S1 under Task 1, in which the time has been normalized. The elbow angle is large initially and gradually decreases and reaches the minimum while the wrist angle increases first and then decreases. The angle changes of the shoulder I and III are similar to the elbow, and shoulder II is similar to the wrist. This is consistent with our daily activity experience. The coordination quantification of the upper limb can be reflected by the movement data, based on which we can extract the coordination features and generate coordinated elbow–wrist trajectories. Figure 11 gives an example of the coordination relationship trained from the CNN-LSTM model.

**Figure 12:**
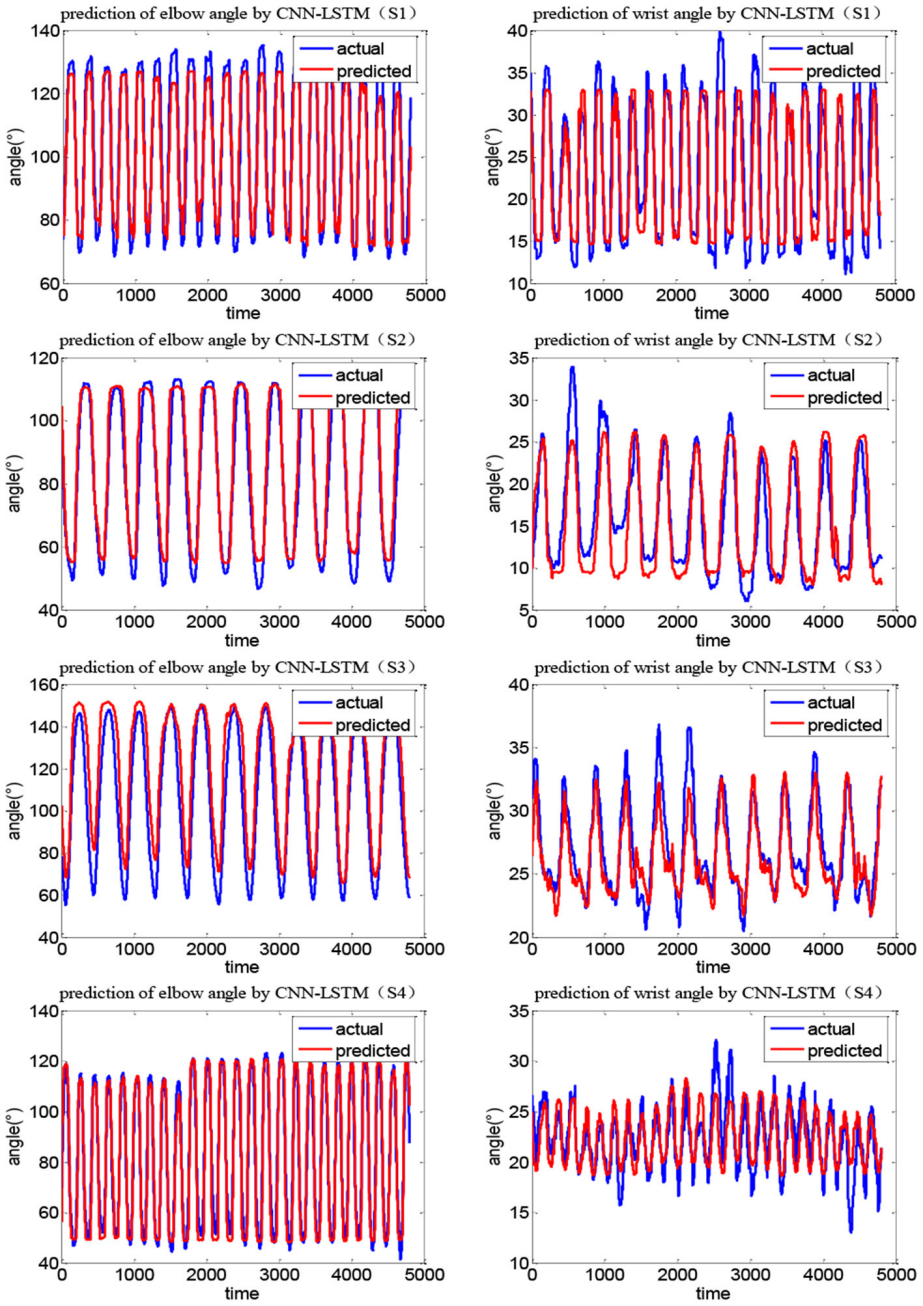
Joint angle prediction results of four subjects (Task 1).

**Figure 13:**
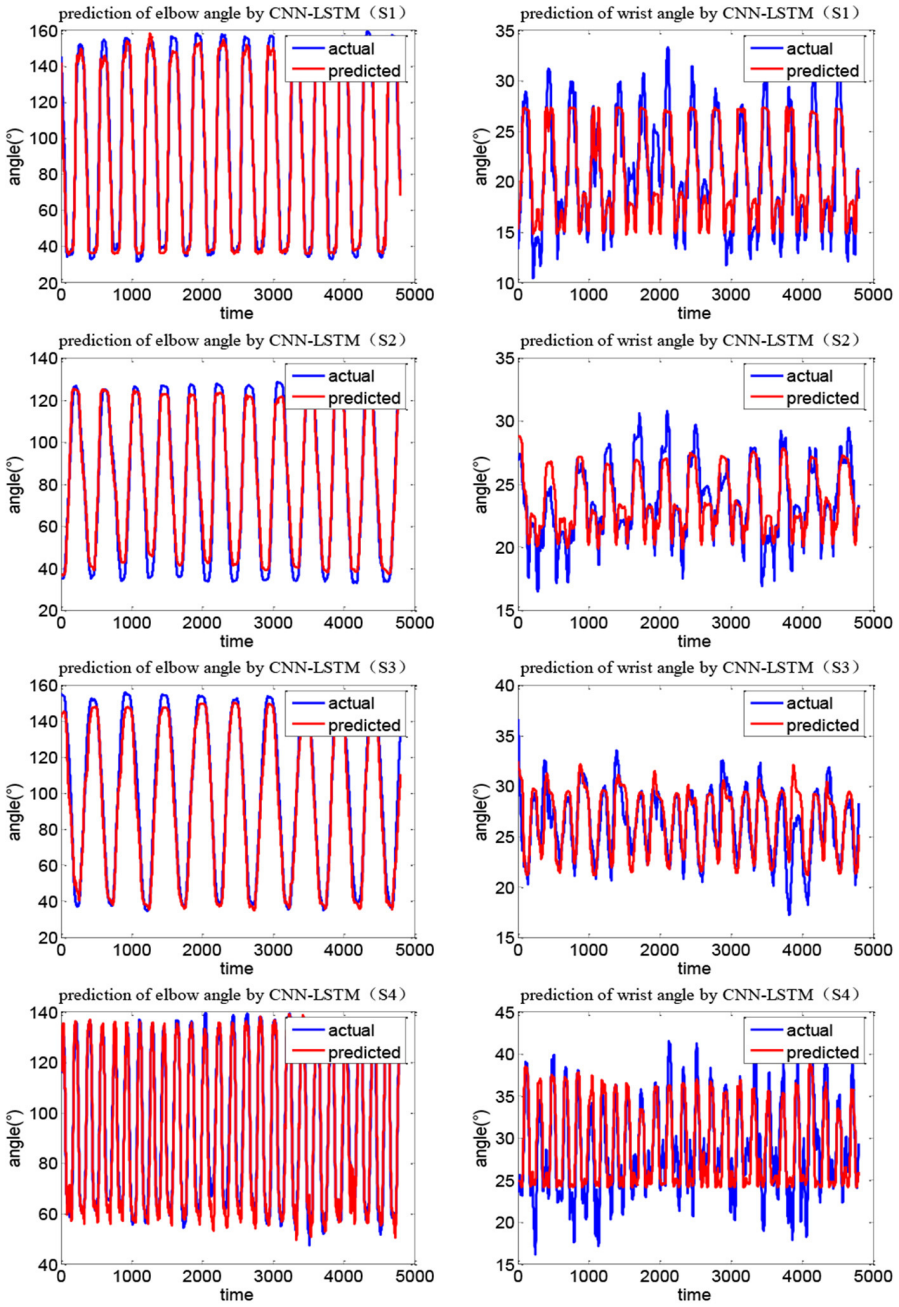
Joint angle prediction results of four subjects (Task 2).

Figures 12, 13 show the joint angle predicted results of different subjects in Task 1 and Task 2, respectively. From the results, though the actual recorded trajectories were easier to obtain and were useful for certain fixed tasks, they have limited effects on the coordinated elbow and wrist training. First, the movements of the elbow and wrist joints of stroke patients are usually abnormal (Bilić et al., 2001; Squeri et al., 2014), so we cannot obtain the recorded trajectories. Even if the elbow–wrist trajectories of the patients are recorded, the coordination between these joints cannot be guaranteed. Thus, a prediction model is required to generate coordinated paths by imitating the movements of normal people. The recorded trajectories of different upper limbs for a specific task are similar in tendency, due to the differences among subjects with various physical parameters or recovery stages, the coordination trajectories are different among subjects. While the adaptability and personality of the coordinated paths can be ensured by the CNN-LSTM using the shoulder data of the patient. The elbow angle predicted by CNN-LSTM has a high coincidence with the actual angle and the results of four subjects with two tasks verified this. In addition, the recorded paths can only suit certain tasks while the quantification model has potential for other tasks.

**Figure 14:**
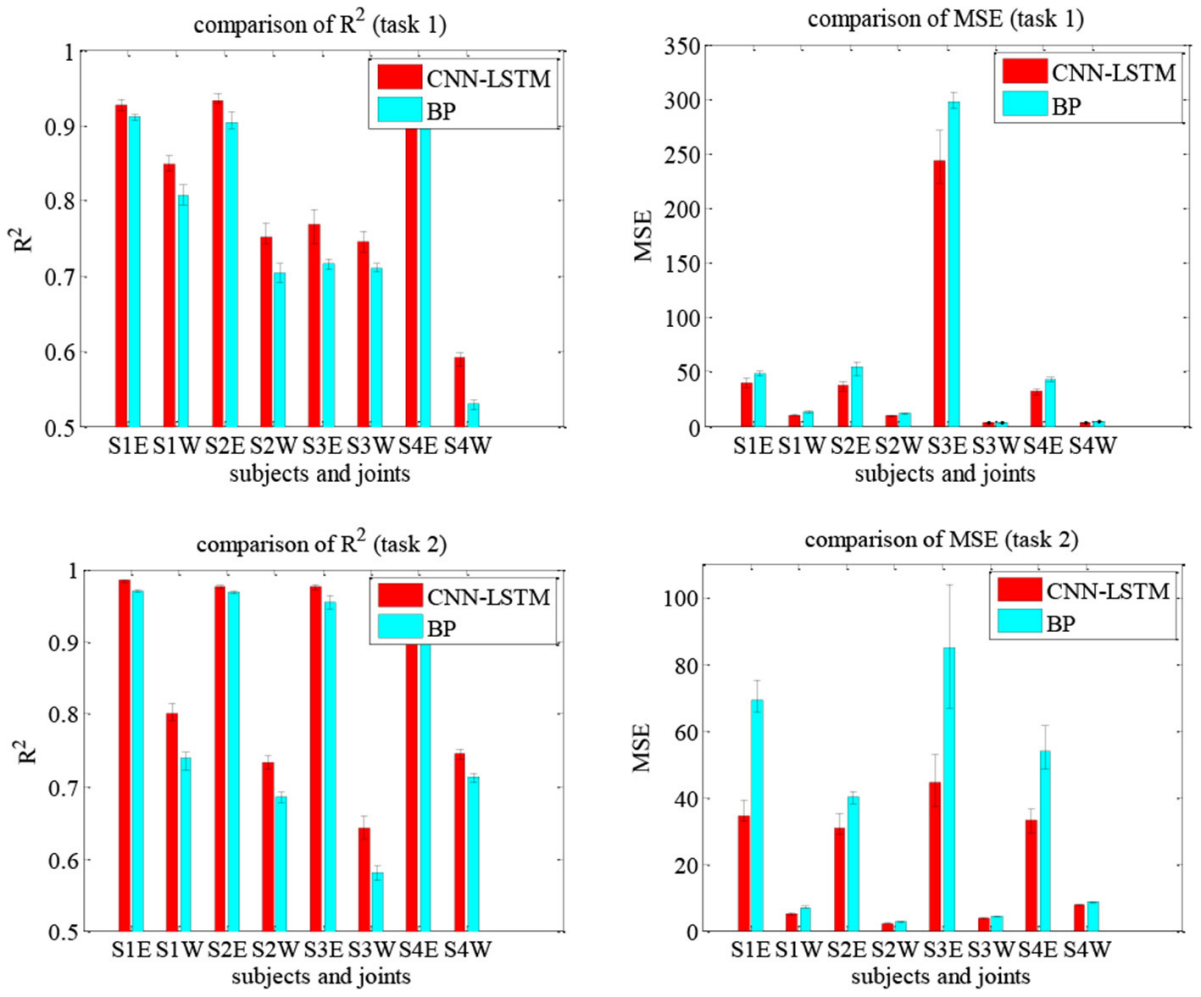
Comparison of the effectiveness of the upper limb joints prediction using the CNN-LSTM and BP models under Tasks 1 and 2.

The current coordination trajectories tend to use pre-defined analytic expressions to describe the coordination of the elbow and wrist joints (Soltani et al., 2017; Ballesteros-Escamilla et al., 2019). However, pre-defined coordination trajectories, similar to recorded trajectories, cannot adapt to different subjects or tasks. In addition, the analytic methods usually plane trajectory in the task space and then calculated the joint motions, which cannot fully reflect the coordination in joint space. In comparison, the CNN-LSTM can be used to directly quantify the coordination relationship in the joint space and possesses adaptability to different patients. Compared with the back propagation (BP) model, the CNN-LSTM model was evaluated by R^2^ and mean square error (MSE). Figure 14 shows the results of two network models for Tasks 1 and 2, in which S1E represents the elbow of subject S1 and S1W the wrist. It can be seen that the *R*^2^ of CNN-LSTM is greater than that of BP for both tasks and has smaller MSE errors. Taking subject S1 in Task1 as an example, based on the CNN-LSTM model and BP model, the average *R*^2^ of the elbow joint was 0.926 and 0.911, respectively, while the wrist was 0.848 and 0.807. When using the CNN-LSTM and BP models, the average MSE of the elbow was 39.842 and 47.834, while the wrist was 10.492 and 13.398, respectively. Considering the difference in the range of motion, the errors were normalized. The relative elbow errors of subject S1 in Task1 are 0.007 and 0.009, respectively.

### Coordinated Control

**Figure 15:**
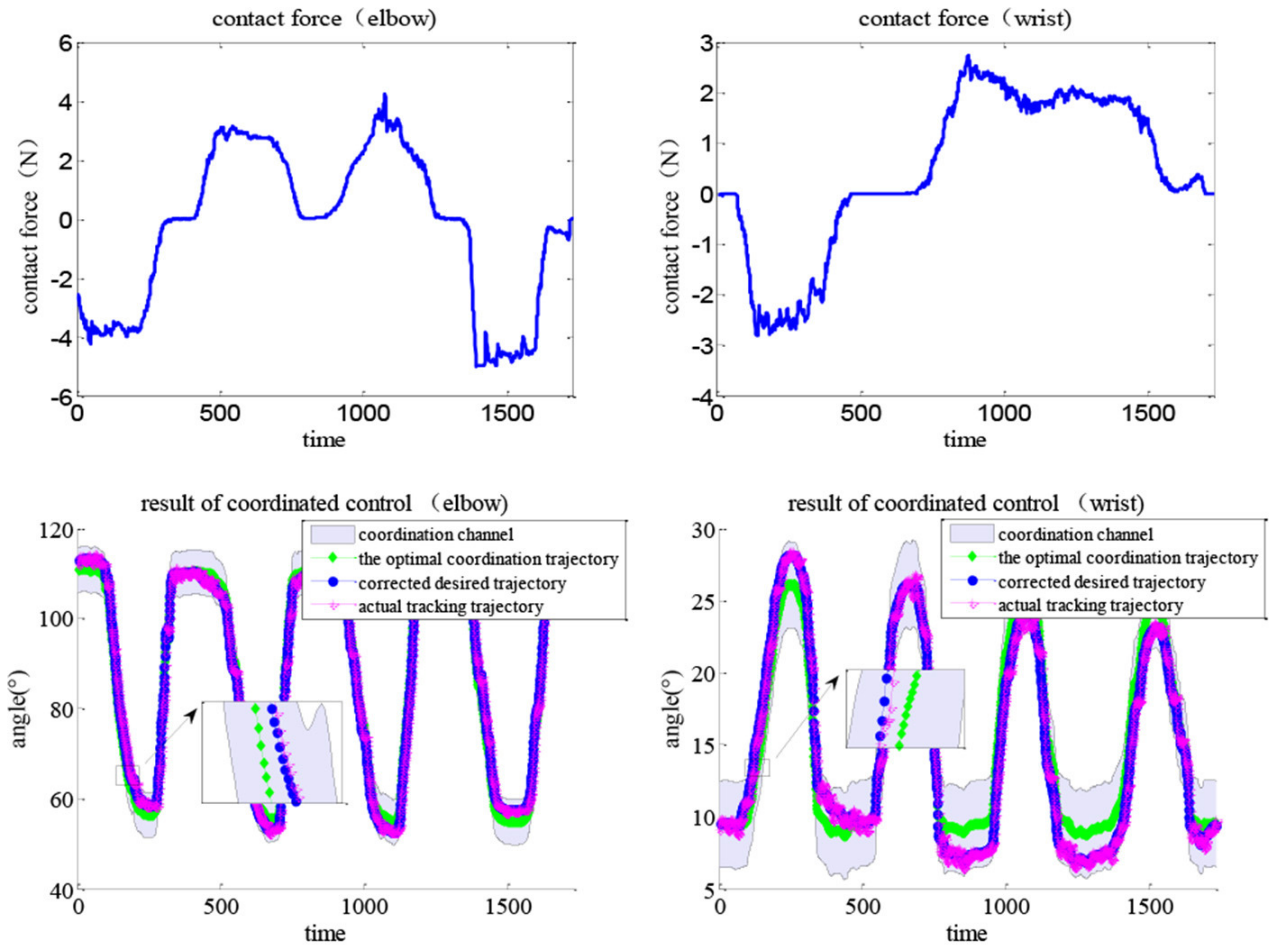
Results of the coordination control with human contact force under Task 1, lines indicating the coordination channel, the optimal trajectory, the corrected trajectory (*via* impedance control), and the actual tracking trajectory (*via* MISO-IMFAC).

An APF-based impedance adjustment scheme was proposed for the coordinated control of the soft elbow–wrist exoskeleton. Each subject was asked to wear the elbow–wrist exoskeleton to carry out coordinated experiments with the two tasks. The optimal coordination trajectories of elbow and wrist joints were generated by the CNN-LSTM model. Considering the range of the position control and the requirement of the joint coordinated motions, the width of the elbow and wrist coordination channels were set to 10° and 6° in Task 1, and 10° and 3° in Task 2, respectively. Figure 15 shows the coordinated control results in Task 1, in which the impedance effects were reflected by comparing the contact force with the resulted training paths.

**Figure 16:**
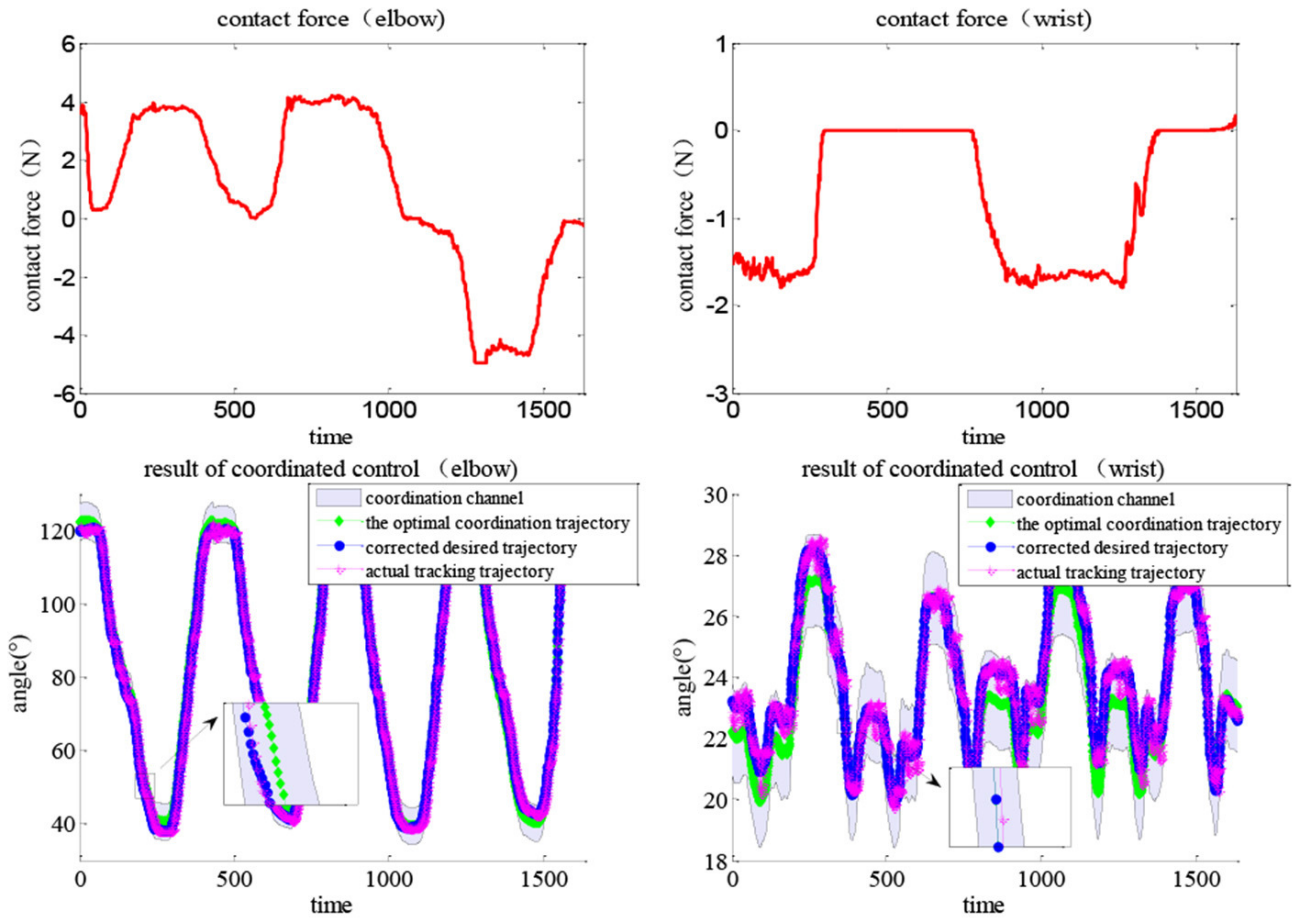
Results of the coordination control with human contact force under Task 2, lines indicating the coordination channel, the optimal trajectory, the corrected trajectory (*via* impedance control), and the actual tracking trajectory (*via* MISO-IMFAC).

During movement, the subjects can exert arbitrary forces on the exoskeleton robot. The human contact force (top line) directly corrects the optimal trajectory *via* adaptive impedance control and the actual path (bottom red line) can timely track the desired (bottom blue line) *via* the MISO-IMFA control, with a partially enlarged view to show the results more clearly. As MISO-IMFAC is a data-driven control method and does not rely on the system model, the PAM hysteresis caused by the change in impedance or admittance did not have a large impact on the control response. Similarly, the experimental results of Task 2 are shown in Figure 16, in which the corrected and actual trajectories were constrained to the virtual coordinated channel. With the human active interaction, the original trajectory was corrected by the adaptive impedance controller. The adopted APF method regards the boundary of the coordinated channel as an obstacle and the optimal trajectory as the target to generate a virtual channel in space. The impedance control based on APF adjustment can correct the desired trajectory according to the active intention of the patient and always constrain it within the scope of the coordination channel to ensure rehabilitation safety and effectiveness. Consequently, both elbow and wrist joints were within the coordination channel and the exoskeleton adaptively assisted subjects to complete the interactive coordinated training tasks.

**Figure 17:**
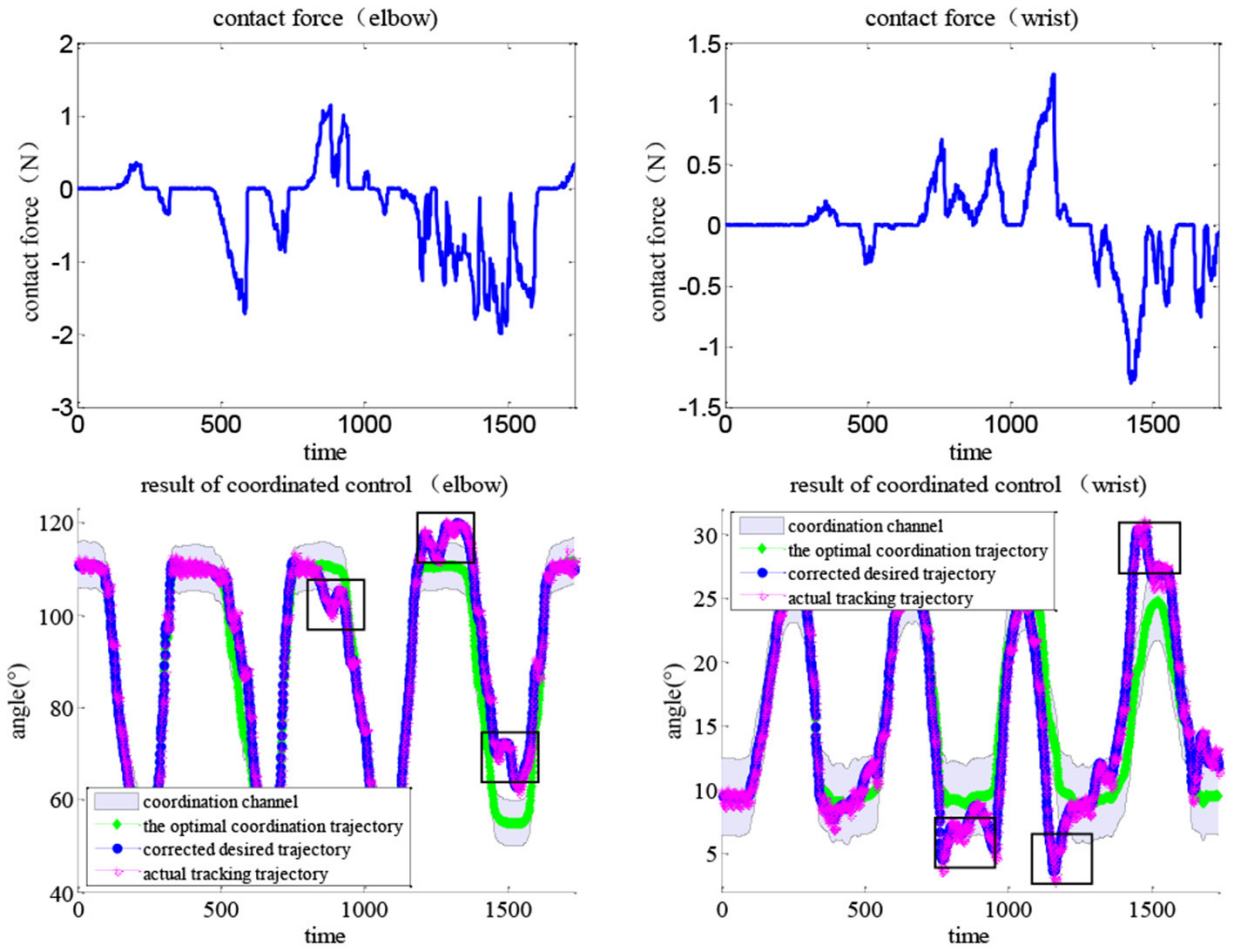
Control results without impedance adjustment under Task 1, the actual trajectory might be out of the coordination channel.

To verify the superiority of the proposed coordinated control method, a comparative experiment was carried out under Task 1. Compared with the common impedance controller with fixed parameters, the results shown in Figure 17 indicate that the actual trajectory might be out of the range of the coordination channel, e.g., the path in the black rectangle. As the contact force increases, the actual trajectory approaches the boundary and finally exceeds the range. In comparison, the robot under the proposed impedance control can always move within the coordination channel, as the parameters are adaptively tuned by the APF scheme, which can drive the robot to move toward the optimal path and away from the boundary. The robot movement can be compliantly and interactively corrected by human contact force while always in a virtual tunnel to keep coordination with the joint space, thus the rehabilitation can be conducted within the coordination channel while maintaining the active motivation of the patient and ensuring movement coordination and safety.

## Discussion and Conclusion

Robot-assisted upper limb training can certainly enhance rehabilitation efficiency and safety, which is important for the recovery of stroke patients. The purpose of coordinated control is to ensure that the joints of the patients can cooperate with each other to complete a delicate task. The coordinated control of the rehabilitation robot can help realize the coordinated training of the upper limb of patients, so how to identify coordination relationships needs to be tackled. Extracting knowledge of the coordination of human joints during movement and applying this relationship to the coordinated control can improve the effectiveness of rehabilitation (Dounskaia et al., 2020). Meanwhile, the modular design makes the robot expandable for different rehabilitation purposes and reconfigurable for various users. When the robot modules are coordinately controlled, the rehabilitation training will become more physiologically meaningful.

Considering the limitations of quantification in the task space, we adopted the quantitative method in the joint space. Two kinds of coordinated tasks were designed, and the data of the shoulder, elbow, and wrist of four subjects were collected by a motion capture system. The angle changes of the upper limb joints under different tasks are obtained, to generate the patient-specific coordinated trajectories. Though the tendency of angle change is similar when completing the same task, there are big differences among subjects in terms of the trajectory range and speed, so the CNN-LSTM model was designed to generate patient-specific coordination trajectories to be determined by the uniqueness of an individual. In the late stage of rehabilitation when the shoulder joint of the patient has returned to normal, one's own shoulder data will be taken into account to generate personalized and coordinated paths. The value of the CNN-LSTM algorithm is reflected by its ability to quantify the coordination of the joints of different subjects so that the generated coordination trajectories are more physiologically meaningful, and also enhances the adaptability of the trajectory to different patients. The effectiveness and advantages of the CNN-LSTM method were presented by comparing it with the BP algorithm and other related studies (Deng et al., 2020; Li Z. et al., 2020). Results show that CNN-LSTM can generate higher correlation coefficients for both the tasks and four subjects, indicating its better performance. The exercise sets can be specific ADL tasks to improve independent living abilities, and CNN-LSTM can guarantee the adaptability of the trajectory to different patients.

An impedance adjustment strategy was then proposed and a coordinated controller including an impedance/admittance model was designed for the modular robot. Different from previous studies, the impedance control was realized on the elbow–wrist exoskeleton within a coordinated channel to ensure the coordination and compliance of the rehabilitation training. Path planning based on coordination relationship ensures that the robot is always in the virtual coordination channel. The impedance adjustment scheme based on APF was designed to correct the original coordination trajectory according to the active intention of the patient and always constrain it within the scope of the coordination channel. The proposed adaptive impedance controller can keep the final movement path within the coordination channel by adopting the APF tuning scheme to ensure the safety and effectiveness of the coordinated rehabilitation. The effectiveness and advantages of the APF-based impedance control method were presented by comparing it with common impedance controller and some existing studies (Erol and Sarkar, 2008; Li et al., 2019a,b), results showed that the path under common methods might be out of the coordination channel, while the robot under the proposed impedance control can always move within the coordination channel corrected by the human contact force with the parameters adaptively tuned by the APF scheme. Consequently, the rehabilitation can be conducted while maintaining the active motivation of the patient and ensuring movement coordination and safety.

In this study, a CNN-LSTM model was designed to plan coordinated trajectories using data from specific ADL tasks, and an APF-based adaptive impedance controller was proposed for coordinated training within a virtual tunnel to meet the personalized training needs. This work can accurately plan the patient-specific coordination trajectory of the elbow and wrist joints and then correct the actual path according to the active intention of the patient within a coordination channel. The MISO-IMFAC method was adopted to realize the position tracking control of the soft modular exoskeleton. A quantification method was proposed for multi-joint coordination to represent the interrelationship of the upper limb during movement, and a novel coordinated control method in cooperating adaptive impedance model of the elbow–wrist exoskeleton was proposed for coordinated training. The experimental results showed that the CNN-LSTM model can quantify the joint relationship and predict the elbow and wrist joint data well, which is verified on four different subjects with two representative ADL tasks, i.e., drinking water and touching the head. During the coordinated control, the initiative of the subject was retained and the quantitative coordinated relationship can be reflected by the planned path. With the robot assistance, the subject can move around the coordinated trajectory within the virtual channel, ensuring training safety and coordination.

The limitations of this work include that we just verified the effectiveness of the proposed method on healthy subjects and the patients with elbow–wrist disorders but normal shoulder movements will be recruited and the results will be reported in the near future. Only two typical ADL tasks were selected for the coordination quantification and coordinated control experiment. The CNN-LSTM model with adaptability can potentially be used on more ADL tasks, but we have not verified it in this study. In the future, we will add other rehabilitation tasks to further verify the adaptability of the model and to make rehabilitation training more diverse. The CNN-LSTM model design can also be further improved by integrating with patient-specific parameter settings and structure optimization. In addition, physiological signals can accurately reflect the intention of the patient, so the combination of electromyogram (EMG) and electroencephalogram (EEG), with the robot is also the focus of the next stage work.

## Data Availability Statement

The original contributions presented in the study are included in the article/Supplementary Material, further inquiries can be directed to the corresponding author/s.

## Ethics Statement

The studies involving human participants were reviewed and approved by Ethics Committee of Wuhan University of Technology. The patients/participants provided their written informed consent to participate in this study.

## Author Contributions

QL, YLiu, and YLi participated in the design of methods, data collection and analysis, and drafted the manuscript. WM participated in the design of the method and participated in the idea selection. CZ helped in the data extraction and manuscript revision. QA and SX participated as a supervisor and modified the manuscript. All authors read and approved the final manuscript.

## Funding

This work was supported by the National Natural Science Foundation of China (52075398), the Research Project of Wuhan University of Technology Chongqing Research Institute (YF2021-17), and the Application Foundation Frontier Project of Wuhan S&T Program (2020020601012220).

## Conflict of Interest

The authors declare that the research was conducted in the absence of any commercial or financial relationships that could be construed as a potential conflict of interest.

## Publisher's Note

All claims expressed in this article are solely those of the authors and do not necessarily represent those of their affiliated organizations, or those of the publisher, the editors and the reviewers. Any product that may be evaluated in this article, or claim that may be made by its manufacturer, is not guaranteed or endorsed by the publisher.
